# Development of chimeric multivalent proteins for serological diagnosis of African animal trypanosomosis

**DOI:** 10.1017/S0031182025100747

**Published:** 2025-10

**Authors:** Robert Eustache Hounyèmè, Loïc Rivière, Antoine Abel Missihoun, Veerle Lejon, Akpole Koffi Kouakou, Christina Calmels, Zakaria Bengaly, Casmir Akpovi, Souaibou Farougou, Sophie Thévenon, Dramane Kaba, Alain Boulangé

**Affiliations:** 1 Unité de recherche et laboratoire de trypanosomiase et leishmaniose, Institut Pierre Richet, Bouaké, Côte d’Ivoire; 2 Département de Génétique et des Biotechnologies, Faculté des Sciences et Techniques (FAST), Université d’Abomey-Calavi, Cotonou, Bénin; 3 INTERTRYP, Université de Montpellier, CIRAD, IRD, Montpellier, France; 4 CIRAD, UMR INTERTRYP, F-34398 Montpellier, France; 5 Microbiologie Fondamentale et Pathogénicité, Université de Bordeaux, CNRS UMR 5234, Bordeaux, France; 6 Unité de recherche sur les maladies à vecteurs et biodiversité, Centre International of Recherche-Développement sur l’Elevage en zone Subhumide (CIRDES), Bobo-Dioulasso, Burkina Faso

**Keywords:** African animal trypanosomosis, chimeric multivalent antigen, diagnosis, genetic engineering, *Trypanosoma brucei*, *Trypanosoma congolense*, *Trypanosoma vivax*

## Abstract

The control of African animal trypanosomosis (AAT) relies on accurate diagnostic tools. Serological diagnosis using ELISA is well-suited for surveillance due to its high-throughput capacity, low cost, and adaptability to rapid formats. However, the WOAH-recommended antibody ELISA for AAT, based on trypanosome lysates purified from rodent blood, can lack specificity and presents standardization challenges as well as ethical concerns. Recombinant proteins offer a solution to standardization, often improving specificity, though potentially at the expense of sensitivity. Combining multiple recombinant proteins can enhance sensitivity while maintaining specificity. Therefore, this study developed chimeric proteins for serological diagnosis of AAT, composed of highly immunoreactive regions from multiple known antigens using genetic engineering. Following an inventory of immunodominant antigens, we selected candidates and, using bioinformatics, designed five chimeric constructs *in silico*: three species-specific and two pan-trypanosome. The coding sequences for these chimeras were synthesized, cloned into expression vectors, and expressed in *Escherichia coli*. Purification was achieved through a series of chromatographic steps, including Ni-NTA affinity chromatography, Q Sepharose ion-exchange chromatography, and Sephadex 200 size-exclusion chromatography. Preliminary assessment of their reactivity with sera from cattle experimentally infected with *Trypanosoma vivax, T. congolense* or *T. brucei* yielded promising results. Longitudinal analysis comparing their reactivity with trypanosome lysates revealed that those specific to *T. congolense*, and *T. vivax*, as well as one pan-trypanosome, can discriminate pre- and post-infection sera. These observations confirm the potential of our chimeric constructs. Future work will involve evaluating these chimeras against a broader panel of sera.

## Graphical abstract

**Figure:**
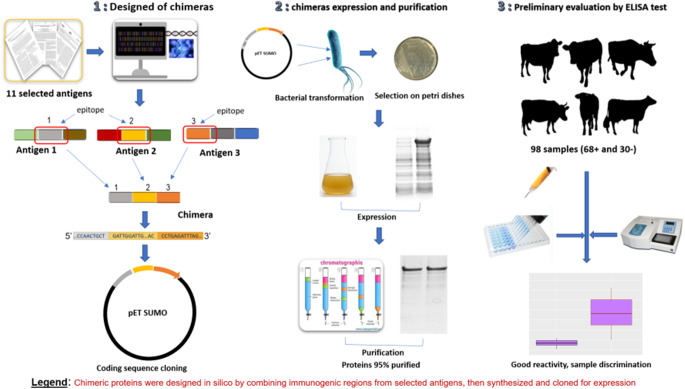

## Introduction

Trypanosomoses are parasitic diseases that can have devastating effects if left uncontrolled. These zoonotic diseases are caused by parasites of the genus *Trypanosoma*, which are primarily transmitted by hematophagous flies of the genus *Glossina* (Alsan, 2015), also known as tsetse flies, but also for some *Trypanosoma* species by mechanical transmission (Desquesnes and Dia, 2004). The diseases affect both humans (Human African Trypanosomiasis: HAT, also known as sleeping sickness) and animals (African Animal Trypanosomosis: AAT, or *nagana*).

The acute form of HAT, caused by *Trypanosoma brucei rhodesiense*, is found in East and Southern Africa, while the chronic form, caused by *T.b. gambiense*, is found in West and Central Africa. Once epidemic, sustained control has led to the elimination of human African trypanosomiasis as a public health problem, with the goal of eliminating transmission of gambiense HAT by 2030 (Lejon et al. 2025). Conversely, African animal trypanosomosis (AAT), caused by *T. congolense* and *T. vivax*, and to a lesser extend *T. brucei brucei*, is arguably one of the most important livestock diseases in Africa. These parasites are mainly found in sub-Saharan Africa, although *T. vivax*, which can be transmitted mechanically, has spread to South America (Davila et al. 1998), and more recently been reported in the Middle East (Asghari and Rassouli, 2022). AAT leads to a strong reduction in livestock productivity (Namangala and Odongo, 2014) which has a significant economic impact on millions of people in sub-Saharan Africa (Kristjanson et al. 1999).

In the absence of vaccine, combating AAT relies on vector control, chemotherapy and the breeding of trypanotolerant cattle. In order to effectively treat the disease, however, the infection must first be detected. This requires diagnostic tools that are simple, accurate, inexpensive and adapted to limited resources (the ASSURED and REASSURED approach) (Land et al. 2019). Diagnostic methods for AAT, whether clinical, parasitological or molecular, all present limitations. Clinical signs are not specific to the disease, parasitological diagnosis suffers from low sensitivity, and molecular tests are often ill-suited for resource-limited settings. Immunological tests and rapid formats are well-suited for surveillance campaigns. Their rapid performance enables immediate mass screening, and their simplicity allows use by non-specialist personnel. Furthermore, they provide valuable epidemiological data for refining control strategies, though potential limitations in sensitivity and specificity must be considered (Tounkara et al. 2021a).

The ELISA test currently recommended by the World Organization for Animal Health (WOAH, founded as OIE) is an antibody detection test based on whole trypanosome lysates produced *in vivo*. This test allows for high-throughput analysis (Desquesnes et al. 2022), however, despite a good sensitivity, it has major limitations. Indeed, preparation of the test reagents involves purifying live trypanosomes from infected-rodents blood by ion-exchange chromatography on DE-52 cellulose (Ekejindu et al. 1986). Besides the ethical concerns associated with the slaughter of a large number of laboratory animals, the resulting whole parasite lysates may prove difficult to standardize due to their complexity and instability. Scaling up production in view of commercialization is although a challenge. Some of the shortcomings could be alleviated in the frame of the COMBAT project (Boulangé et al. 2022) whereby *in vivo*-produced parasites were substituted by *in vitro* cultured trypanosomes (Bossard and Desquesnes, 2023). This came as an improvement by avoiding the ethical concerns of *in vivo* production and ameliorating the standardization of antigen production. The problems of specificity inherent to such a complex antigen, the cross-reactivity between species generating occasional display a high false positive rate (Hounyèmè et al. 2022), and the limited possibilities of scaling up production, still remain.

An alternative is the use of recombinant proteins. The diagnostic performance of a number of recombinant antigens has been evaluated over the years, and two of them, GM6 (Pillay et al. 2013) and TcoCB1 (Mendoza-Palomares et al. 2008) have been used to develop a semi-commercial rapid diagnostic test (Boulangé et al. 2017). Although recombinant proteins eliminate the problems of standardization and ethical considerations, and generally tend to increase specificity, they often do so at the expense of sensitivity, linked to the very limited number of epitopes that can be displayed by a single protein (Tounkara et al. 2021b; Desquesnes et al. 2022).

Recent studies have shown that the combination of several recombinant proteins could increase sensitivity while maintaining high specificity (Tounkara et al. 2021b). Nonetheless, a mix of recombinant proteins still stumbles upon the issue of standardization. An alternative approach is the use of recombinant multivalent chimeric constructs, composed of the antigenic determinants of different antigens. This approach has proven its worth in the diagnosis of a number of diseases, both human and animal, including parasitic infections, as was recently reviewed by (Gonçalves et al. 2024), referring to recombinant multiepitope proteins (RMPs) rather than chimeras. The authors states that a higher degree of sensitivity and specificity is achieved due to the high density of epitopes within the RMPs. Concerning Trypanosomatidae, for Chagas’ disease and leishmaniasis, the specific challenges associated with test standardization could be overcome using chimeras, while improving diagnostic accuracy, particularly for the chronic forms of these diseases when immune responses are often more complex to detect (Santos et al. 2016, 2017, 2020). In addition, (Aguirre et al. 2006) investigated in *T. cruzi* infections how chimeric proteins compare with peptide mixtures, and could confirm their superiority in terms of sensitivity and specificity, and reduction of cross-reactions.

Building upon the promising performance reported in previous studies, we evaluated the application of multivalent recombinant antigens for the serological diagnosis of African trypanosomosis.

## Materials and methods

### Design of the chimeras coding sequences

**Figure 1.**
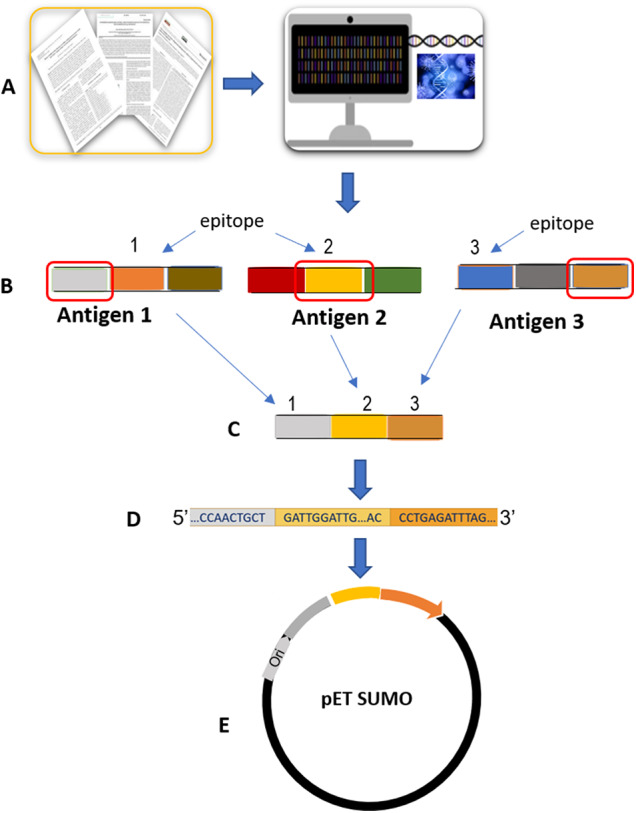
Steps from chimera design to coding sequence cloning. A: systemic literature review and comprehensive bioinformatics analysis, B: selected epitopes/regions, C: construction of chimera, D: optimized coding sequences, E: coding sequence cloning.

Candidate antigens for the serological diagnosis of African trypanosomosis were identified through a combined approach of systemic literature review and comprehensive bioinformatics analysis (Figure 1).

A thorough literature review was conducted to pinpoint antigens with established diagnostic utility. PubMed and Google Scholar were searched using keywords such as ‘*Trypanosoma* diagnostic antigens’, ‘serological diagnosis Trypanosomiasis’, ‘recombinant antigens *Trypanosoma*’, and ‘ELISA recombinant antigen diagnostic’. Selected articles were evaluated based on the diagnostic performance of the antigens, particularly their sensitivity and specificity, to optimize candidate selection. Antigens were prioritized if they were expressed recombinantly, exhibited strong immunoreactivity against sera from target species infections, and, when available, demonstrated a rapid decline in antibody titers post-treatment – a crucial criterion for minimizing persistent reactions in cured animals.

Gene and protein sequences of selected antigens were retrieved from TryTrypDB (https://tritrypdb.org/tritrypdb/app) and Uniprot (https://www.uniprot.org/) and GenBank databases, including strain information and, when available, three-dimensional (3D) protein structures. Sequence conservation analysis was performed to confirm their presence across pathogenic trypanosome species.

Bioinformatics tools were employed to identify highly immunoreactive regions and epitopes. The 3D structures of proteins that were not available were predicted using the AlphaFold2 Colab program and visualized using the ChimeraX software to facilitate identification of their most exposed surface regions. The information obtained was cross-referenced with the Bepipred Linear Prediction 2.0 algorithm (http://tools.iedb.org/main/bcell/) for B-cell linear epitope prediction to validate antigenic potential. Furthermore, BLAST searches identified orthologous sequences of the proteins in trypanosomes and hosts, primarily cattle, to ensure specificity and minimize cross-reactivity.

The generated data enabled us to select the most immunogenic and antigenic regions to be incorporated into the five chimeras. The selected regions were joined end-to-end to construct three chimeras specific for *T. brucei, T. congolense* and *T. vivax*, and two pan-trypanosome chimeras. The 3D structure of the five chimeras was predicted (AlphaFold2 Colab). Finally, the coding sequences were generated *in silico* and codon-optimized for expression in *Escherichia coli.* Sequences were submitted to GenBank with the accession numbers PV171107, PV171108, PV171109, PV171110 and PV171111.

### Expression of chimeric proteins

The coding sequences of the chimeric constructs were synthesized chemically by ProteoGenix (Schiltigheim, France) and provided cloned into the pET-SUMO plasmid vector with a N-terminal histidine tag to facilitate protein purification by affinity chromatography, using the HindIII and XhoI restriction enzyme sites.

Bacterial transformation was carried out according to a protocol adapted from (Nallamsetty and Waugh, 2007). Competent bacteria of the T7 express LysY strain (New England Biolabs, Ipswich, MA, USA) were transformed with the plasmids carrying the chimeric coding sequences. Transformants were selected on Luria-Bertani (LB) agar plates supplemented with kanamycin (50 µg/mL) and incubated at 37°C. Single colonies were then pre-cultured in 5 mL liquid LB medium containing 50 µg/mL kanamycin. After overnight incubation at 37°C, the bacterial preculture was diluted 1:50 in fresh liquid LB and incubated at 30°C. Protein expression was induced with 1 mM isopropylthio-B-D-galactoside (IPTG, Ref: EU0008-B, Euromedex, Souffelweyersheim, France) when the OD_600_ reached 0.6-0.8. Cells were grown at 25°C with constant agitation for four hours and then harvested by centrifugation (4700 g for 20 min at 4°C). The cell pellet was stored at −80°C. To ensure that expression was effective, pre- and post-expression aliquots were prepared and analysed by SDS-PAGE.

### Purification of chimeric proteins

Protein solubility was first checked by recovering cell pellets in lysis buffer (50 mM Tris-HCl pH 8.0, 400 mM NaCl, 7 mM Chaps, 100 µg/mL lysozyme) containing a commercial protease inhibitor mix (Abcam, ref ab274282, Cambridge, UK) and Phenylmethylsulfonyl fluoride (PMSF). After overnight storage at −20°C, the protein lysates were thawed and subjected to sonication on ice, consisting of five two-minute cycles, amplitude 50%, with a three-minute pause between each cycle. Soluble and insoluble fractions were analysed by SDS-PAGE after centrifugation of the samples at 20 000 g for 40 min at 4°C.

Chimeric proteins were purified successively on three different types of columns. First, they were purified by affinity chromatography on nickel resin making use of their 6xHis tag. About 2.5 mL of HisPur^TM^ Ni-NTA resin suspended in 20% ethanol was used for lysates produced from a 500 mL culture. Briefly, the beads were first equilibrated with five column volumes of column buffer (50 mM Tris-HCl pH 8.0, 400 mM NaCl, 10 mM Imidazole). The soluble protein-containing fraction was supplemented with 10 mM Imidazole and then incubated with the beads for one hour at 4°C with gentle agitation before loading onto a column. The column was then washed with 10 column volumes of column buffer. The protein was eluted with five column volumes of column buffer supplemented with 300 mM imidazole, collected in 2 mL fractions and stored on ice. Aliquots were taken at every step for analysis by SDS-PAGE and all samples were then stored at −20°C. The whole process was carried out in a cold room to minimize the risk of protein degradation and/or denaturation.

Protein-containing elution fractions were pooled and supplemented with 2 mM DTT. Carrier removal was performed through enzymatic digestion of the proteins using SUMO protease (1 mg/100 mg protein) through incubation without agitation at 4°C overnight. Successful digestion was confirmed by SDS-PAGE prior to subsequent purification steps.

Purification on HiTrap™ Q and Superdex™ 200 columns was carried out on an ÄKTA pure^TM^ chromatography system (Cytiva, Washington DC, USA). The use of the HiTrap™ Q column for protein purification by ion exchange chromatography relies on the interaction between the charges of the proteins and those of the column resin. Proteins were diluted to a NaCl final concentration of 100 mM to match column equilibration conditions (50 mM Tris-HCl pH 8.0, 100 mM NaCl), and injected into the column. After injection, the column was washed with 12 mL of the same buffer. Elution was performed with an increasing gradient of NaCl (100 mM to 1 M) generated by the ÄKTA system. The collected fractions were analysed by SDS-PAGE and those containing proteins with improved purity selected. Purification of the chimeras was completed by size exclusion chromatography on a Superdex™ 200 column. The column was first equilibrated with a buffer consisting of 50 mM Tris-HCl pH 8, 500 mM NaCl. Fractions selected after the HiTrap™ Q column were pooled and concentrated to a maximum volume of 5 ml, before being supplemented with DTT to 2 mM final, and injected onto the Superdex™ 200 column. Proteins were eluted with 500 mM NaCl, automatically generated by the ÄKTA system. Fractions with the highest purity were identified by SDS-PAGE, pooled, concentrated using a 20 ml Amicon^®^ Ultra device with a molecular cut-off (MWCO) of 30 kDa. Finally, protein concentrations were measured, proteins aliquoted, glycerol added to 10% final, and stored at −80°C.

### Chimera analysis by SDS-PAGE

For SDS-PAGE analysis (Laemmli, 1970), samples were mixed with SDS/β-mercaptoethanol buffer, boiled and migrated onto Mini-PROTEAN TGX 4-20% polyacrylamide gels (Bio-Rad, Hercules, CA, USA) at 200 V for 45 min in Tris-Glycine-SDS buffer. After migration, the gels were stained with InstantBlue Coomassie Protein Stain (Abcam, ref: ab119211) and washed with distilled water. Images were captured using ImageQuant^TM^ LAS 4000 (Cytiva).

### Western blot analysis of chimeric protein recognition by infected sera

To specifically evaluate the ability of antibodies from characterized infected animal sera to recognize the chimeric proteins, we used a strip immunoblotting technique (Western blot). For this, we ran a 4–20% SDS-PAGE gel for each chimera comprising a narrow well (0.3–0.4 cm) for the molecular weight markers and a wide well (6–7 cm) for the protein sample. We loaded 250 µL of each chimera (50 ng/mL) and allowed them to migrate. The protein was then transferred to a PVDF membrane. The membrane was cut into uniform strips and individually blocked with PBST buffer containing 5% skimmed milk for two hours at room temperature. Each serum sample was diluted 1:200 in blocking buffer and incubated with a strip for one hour at 37°C with gentle shaking. After four 5-min washes with PBS containing 0.1% Tween-20, an anti-bovine conjugate diluted 1:5000 in blocking buffer was added and incubated for one hour at room temperature with shaking. A second cycle of four 5-min washes followed. Enzymatic detection of the immune complexes was performed using Pierce™ ECL Western Blotting Substrate (Thermo Fisher Scientific) and chemiluminescence, and the resulting images were captured with the ImageQuant™ LAS 4000 instrument.

For this Western blot validation, we used reference samples including three negative sera, as well as two sera each from cattle infected with *T. congolense, T. vivax* and *T. brucei*. These samples were produced during an experimental infection in cattle aged 12 to 24 months originating from the tsetse-free Dori region of northern Burkina Faso, as described by (Bossard et al. 2021).

### Preliminary evaluation of chimeras by indirect ELISA

The ELISA protocol was adapted from the WOAH Compendium of standard diagnostic protocols for animal trypanosomosis of African origin (https://agritrop.cirad.fr/591960/). Briefly, Nunc™ Polysorp™ (ThermoFisher Scientific) 96-well flat-bottomed plates were sensitized with 1 µg/mL of either chimera. Sera from experimental infections in cattle were used at a 1:100 dilution and secondary antibody (rabbit anti-bovine IgG-HRP, Sigma-Aldrich, St Louis, MO, USA) was diluted 1:20 000. Development was performed using TMB substrate (K-Blue™, Neogen Corporation, Lansing, MI, USA) a stabilized, self-stopping TMB substrate. Optical densities were measured at 650 nm after 30 min, a wavelength appropriate for the substrate’s stable blue chromogen, which does not require an acidic stop solution.

The serum samples used to assess chimera reactivity by ELISA and to detect antibodies against different *Trypanosoma* species were sourced from a separate experimental infection study (currently under publication).

Samples from 24 experimentally infected cattle were used for the detection of antibodies to *T. congolense*. For each animal, the samples included three pre-infection samples and 18 post-infection samples (regular collection frequency noted DPI: Days Post Infection). Samples for the detection of antibodies to *T.b. brucei* were obtained from three experimentally infected cattle and consisted of one pre-infection sample and 13–23 post-infection samples per animal (irregular collection frequency noted TP: Time Point). Finally, to detect antibodies to *T. vivax*, samples from one experimentally infected cow were included one pre-infection sample and 15 post-infection samples.

### Statistical analysis

Data from the evaluation of chimeras with experimental bovine sera were analysed using RStudio software (version 4.2.0). Normal distribution of the data and homogeneity of variances were verified by the Shapiro–Wilk and Bartlett tests, respectively. To assess the reactivity of each chimera, we compared its detection of the target infection with that of the corresponding trypanosome lysate and the other chimeric constructs by performing statistical comparisons of optical densities. The Kruskal–Wallis test was used for overall comparisons between groups. Subsequently, we performed pairwise comparisons using the Dunn’s test with a Benjamini–Hochberg correction (Benjamini and Hochberg, 1995) to specifically assess the discriminatory power with respect to the intended target infection, as well as cross-reactivity.

## Results

### Construction of chimeric sequences

The inventory of antigens with diagnostic potential for AAT and/or HAT in the scientific literature resulted in 66 research papers. Out of those, 24 proteins of interest for our purpose were selected, based on (1) being immunodominant antigens, (2) having been expressed in recombinant systems, (3) having been tested on sera from *Trypanosoma* infections of target species (chiefly bovine) and (4) whose corresponding antibody titers declined rapidly after treatment. Most targeted *T. brucei, T. congolense* or *T. vivax*. Some of the selected proteins detected monospecific trypanosome antibodies while others detected pan-trypanosome antibodies.

Among these 24 proteins, 11 were retained after analysis of their 3D conformational structures and predicted epitopes from which the regions of interest were defined for the construction of chimeras. They include invariant surface antigens (ISG-64 and ISG-75), microtubule-associated proteins (MARP), major glycoproteins (TbbGM6, TcGM6 et TvGM6), proteases (cathepsin B, congopain, vivapain), a heat shock protein (BiP-HSP70), a surface protease (MSP-D) and specific antigens such as TcP46 and MyxoTLm. Most of these proteins were produced in *E. coli*, except for cathepsin B and vivapain, which were expressed in *Pichia pastoris* (Table 1).

**Table 1.** Selected candidates for serological diagnosis

Chimeric antigens size (kDa)	Proteins/sequences names	Expression system	Species specificity	Host	Amino acid range	Gene bank sequence ID	References
*Chimer_Tb* (69.1)	75 kDa invariant surface glycoprotein (ISG75)	*Escherichia coli*	*T.b. gambiense, T. evansi*	Cattle	T^26^-S^290^	Q57VX7	(Tran et al. 2006, Rudramurthy et al. 2015)
	Microtubule-associated repetitive protein (MARP-Tb)		*T.b. brucei*		K^112^-M^149^/ E^378^-E^427^	XP_823257.1	(Müller et al. 1992, Imboden et al. 1995)
	Major glycoproteins, calpain, putative (TbbGM6)		*T.b. brucei, T. evansi, T. equiperdum*	Equine	S^1301^-E^1376^	XP_828202.1	(Verney et al. 2022, Goto et al. 2011)
	64 kDa Invariant surface glycoprotein (ISG64 Tb)		*T.b. gambiense*	Human	S^23^-T^57^/ L^78^-D^95^/ G^121^-E^252^	XP_011773802.1	(Sullivan et al. 2013, Biéler et al. 2016)
*Chimer_Tc* (59.4)	Microtubule-associated repetitive protein (MARP-Tc)		*T. congolense*	Cattle	M^1^-T^156^	CCC94107.1	(Müller et al. 1992, Imboden et al. 1995)
	Major glycoproteins, calpain, putative (TcGM6)				M^1^-A^136^	CCC94721.1	(Goto et al. 2011)
	Cathepsin B-like protease (TcoCB1)	*Pichia pastoris*			S^128^-K^240^/ Y^256^-G^277^	ABY78821.1	(Mendoza-Palomares et al. 2008, Boulangé et al. 2017)
	Cysteine protease precursor (Congopain: CP2)	*Escherichia coli*			P^341^-E^444^	AAA18215.1	(Lalmanach et al. 2002, Authié et al. 1993)
*Chimer_Tv* (62.5)	64 kDa invariant surface glycoprotein (ISG64 Tv)	*Escherichia coli*	*T. vivax*	Calves	S^40^-T^350^	CCD21631.1	(Fleming et al. 2016)
	Major glycoproteins, putative (TvGM6)	*Escherichia coli*		Cattle	D^1^-L^65^/ D^261^-L^325^	CCC52616.1	(Pillay et al. 2013)
	Cathepsin L (Vivapain)	*Pichia pastoris*			V^332^-N^456^	KAH8613465.1	(Eyssen et al. 2018)
*Chimer_PanTryp-1* (76.3)	Putative GTP-binding protein (MyxoTLm)	*Escherichia coli*	*T. vivax*		M^1^-T^159^/G^200^-L^275^	CCC47237.1	(Pinheiro et al. 2021)
	Major glycoproteins, calpain, putative (TbbGM6)		*T.b. brucei*		M^544^-E^620^	XP_828202.1	(Goto et al. 2011)
	Major glycoproteins, calpain, putative (TcGM6)		*T. congolense*		M^1^-R^76^	CCC94721.1	
	Major glycoproteins, putative (TvGM6)		*T. vivax*		D^391^-A^463^	CCC52616.1	(Pillay et al. 2013)
	Heat shock 70 kDa (Bip-HSP70)		*T. congolense*		V^420^-G^644^	CAA80279.1	(Boulangé et al. 2002)
*Chimer_PanTryp-2* (62.5)	Major surface protease (MSP-D)			Mousse	C^133^-K^156^/G^227^-N^260^/E^311^-G^342^/K^434^-A^455^/P^471^-P^497^	CCC95354.1	(Marcoux et al. 2010)
	Heat shock 70 kDa (Bip-HSP70)			Cattle	K^107^-K^129^/K^272^-K^298^/V^373^-A^395^	CAA80279.1	(Boulangé et al. 2002)
	Putative GTP-binding protein (MyxoTLm)		*T. vivax*		D^299^-Y^369^	CCC47237.1	(Pinheiro et al. 2021)
	*Trypanosoma congolense* 46 kDa (TcP46) Hypothetical protein		*T. congolense*	Mousse Cattle	R^151^-K^301^	CCD11626.1	(Zhou et al. 2014)
	Heat shock 70 kDa (Bip-HSP70)			Cattle	T^528^-G^642^	CAA80279.1	(Boulangé et al. 2002)

Based on these antigens, five chimeric proteins were designed *in silico* by stringing together end-to-end the regions containing the immunoreactive epitopes. Three chimeras were specific for each of the three target-species, that is, *T. brucei* s.l. ‘*Chimer_Tb*’ (also referred as *Trypanozoon), T. congolense* ‘*Chimer_Tc*’ and *T. vivax* ‘*Chimer*_*Tv*’, and two were constructed as pan-trypanosome ‘*Chimer_PanTryp-1, Chimer_PanTryp-2*’, that is, supposed to detect an infection by any species of African trypanosomes (Table 1). Their three-dimensional structures were predicted, which is a gauge of proper folding, hence soluble expression (supplementary data).

For proteins MARP and GM6, characterized by repetitive sequences exhibiting minor amino acid variations (one or two amino acids between repetitions), motifs analysis was performed to identify sequences representative of this diversity, and a conjunct of three repeats varying slightly were subsequently used. These motifs from repeated sequences, along with other antigenic fragments, were incorporated into the three mono-specific constructs and into the first multi-specific chimeric construct *Chimer_PanTryp-*1. The generated chimeras comprised between 3 and 11 antigenic regions, sourced from three to five distinct proteins per construct. The spatial arrangement of the fragments within the chimeras was designed to approximate their relative position (N-terminal, internal or C-terminal) within the original proteins. Additionally, closely juxtaposed epitopes were aggregated into consolidated regions to optimize immunoreactivity.

### Expression and purification of chimeric proteins

**Figure 2.**
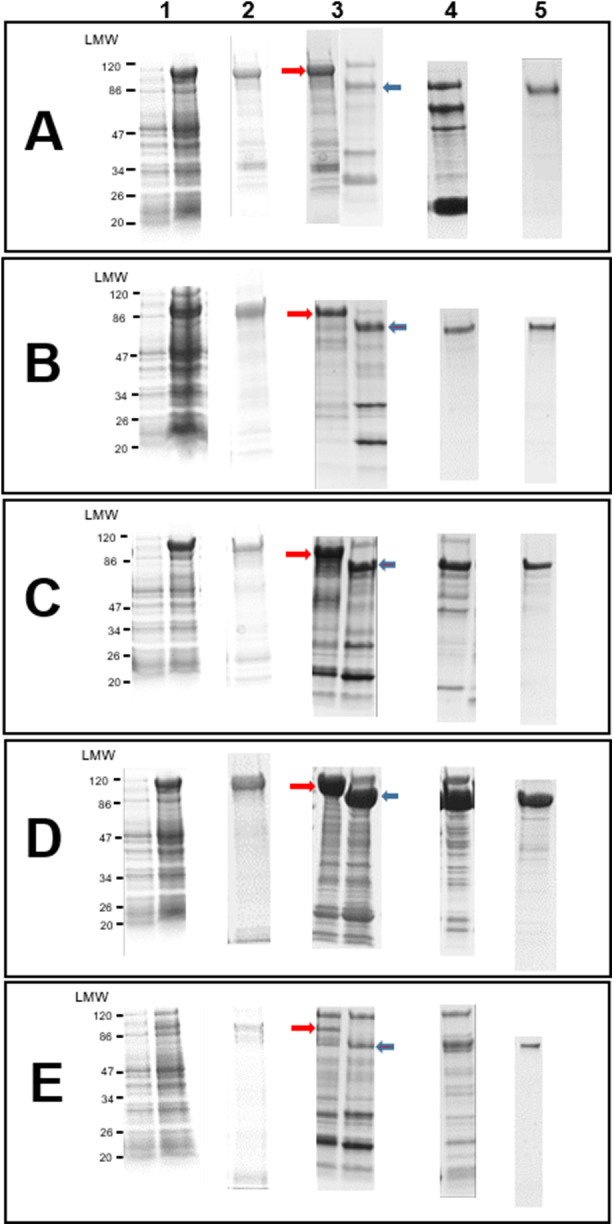
Stepwise expression and purification of chimers. A: *chimer_Tb*, B: *chimer_Tc*, C: *chimer_Tv*, D: *chimer_PanTryp-1*, E: *chimer_PanTryp-2.* 1: expression, 2: Ni-NTA affinity chromatography, 3: digested protein, 4: Q Sepharose ion-exchange chromatography, 5: Sephadex 200 size exclusion chromatography, LMW: low molecular weight.

After optimizing the expression conditions in terms of temperature, duration of bacterial cultivation before and after induction, concentration of the IPTG inducer, buffers composition, and cell lysis conditions, all five chimeric proteins could be expressed as soluble proteins at the expected sizes (Table 1). A purification protocol based on a succession of three types of chromatography, namely affinity chromatography on nickel ions, ion exchange chromatography on Q sepharose and molecular sieve (size exclusion chromatography) on Sephadex 200, coupled with removal of carrier by the SUMO protease, lead to a protein yield ranging from 1 mg/L to 5 mg/L culture, depending on the chimera considered, with an estimated purity exceeding 90% (Figure 2).

### Chimeric protein recognition by antibodies in infected animal sera (Western blot)

**Figure 3.**
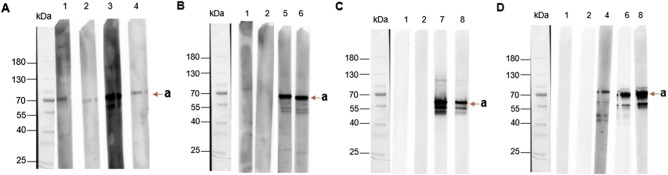
Chimeric protein recognition by antibodies in infected animal sera. A.: *Chimer_Tb*; B.: *Chimer_Tc*; C.: *Chimer_Tv*; D.: *Chimer_PanTryp-1*; kDa: kiloDalton; 1–2: negative sera tested with all chimeras; 3–4: sera from cattle infected with *T. brucei*; 5–6: sera from cattle infected with *T. congolense*; 7–8: sera from cattle infected with *T. vivax*; a: expected size for each chimera.

A comparative analysis of immunoblots reveals a distinct recognition profile for each chimera. For *Chimer*_*Tb*, distinct recognition bands are present at the level expected for the chimera when using the *T. brucei*-positive control samples. However, bands are also observed at the same size with negative control samples. For *Chimer*_*Tc*, no recognition bands are observed for the negative controls, while a clear, intense band is visible for the *T. congolense* positive controls. Likewise, for *Chimer*_*Tv*, negative control samples show no recognition, but distinct, intense bands are detected with *T. vivax* positive control samples. Finally, *Chimer_PanTryp-1* does not show any recognition band with the negative controls, while a recognition band is visible for all positive control samples, including those from *T. brucei, T. congolense and T. vivax* (Figure 3).

### Preliminary evaluation of chimera reactivity and analytical specificity

#### Comparative analysis of the reactivity of chimeras and lysates of trypanosomes (recommended by WOAH)

Evaluation of chimera reactivity revealed distinct profiles depending on *Trypanosoma* species and antigen type. The exception was *Chimer_PanTryp-2*, which showed no reactivity.

**Figure 4.**
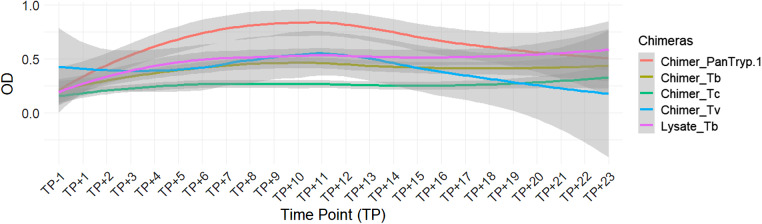
Evaluation of chimera reactivity with bovine sera from experimental *T. brucei* infection. OD: optical density; TP: time point; Lysate_Tb: antigen recommended by WOAH.

The Kruskal–Wallis test showed that, in the case of *T. brucei* infection sera, *Chimer_PanTryp-1* exhibited a rapid increase in reactivity, peaking at around OD 0.9 between the time point (TP) +8 and TP +10, which was higher than that observed for *Chimer_Tb* and the reference Lysate_Tb. There was a statistically significant difference in reactivity between *Chimer_PanTryp-1* and Lysate_Tb (*P* = 0.0126), whereas no such difference was detected between *Chimer_Tb* and Lysate_Tb (*P* = 0.0811). *Chimer_Tb* showed a moderate plateau around OD 0.45–0.5 (Figure 4).

**Figure 5.**
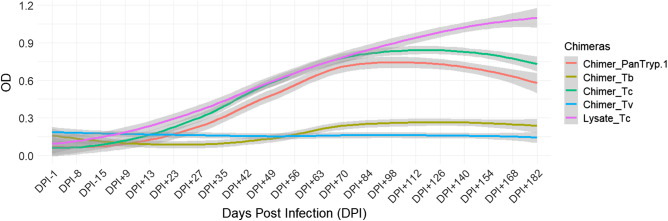
Evaluation of chimera reactivity with bovine sera from experimental *T. congolense* infection. OD: optical density; DPI: days post infection; Lysate_Tc: antigen recommended by WOAH.

Regarding *T. congolense* infection, the reference Lysate_Tc exhibited a gradual and continuous increase in reactivity, surpassing *Chimer_Tc* significantly (*P* = 0.0003) in the advanced chronic phase, reaching levels above 1.1 OD in the long term. The reactivity of *Chimer_PanTryp-1* peaked at around OD 0.75 before declining. This reactivity was significantly lower than that of Lysate_Tc (*P* = 0.0000) (Figure 5).

**Figure 6.**
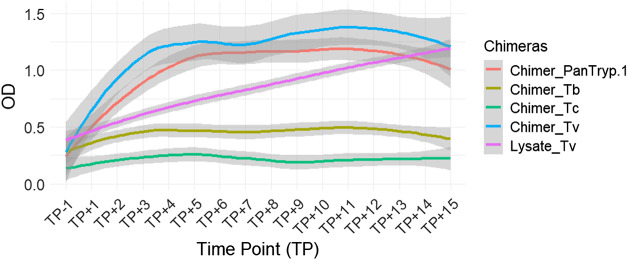
Evaluation of chimera reactivity with bovine sera from experimental *T. vivax* infection. OD: optical density; TP: time point; Lysate_Tv: antigen recommended by WOAH.

For *T. vivax* infection, *Chimer_Tv* demonstrated the highest and fastest reactivity, reaching plateau levels of approximately OD 1.3–1.4. Although Lysate_Tv started at a lower level, its reactivity increased gradually, surpassing that of both *Chimer_Tv* and *Chimer_PanTryp-1* by the end of the follow-up period. Statistically significant differences were observed between *Chimer_Tv* and Lysate_Tv (*P* = 0.0005), as well as between *Chimer_PanTryp-1* and Lysate_Tv (*P* = 0.0005). *Chimer_Tv* and *Chimer_PanTryp-1* exhibited faster kinetics of reactivity. Additionally, a highly significant difference (*P* = 0.0000) was noted between *Chimer_Tv* and *Chimer_PanTryp-1* (Figure 6).

#### Analytical specificity of chimeras: comparison of the target infection versus the other two infections

The specificity analysis of the chimeric proteins was performed by comparing the reactivity of sera from animals infected with the target species to those infected with the two other *Trypanosoma* species. A chimera showing significant differences between these groups could suggest a certain specificity for the target infection.

Overall, chimeras generally showed low and stable reactivity when tested with sera from infections with non-target species, confirming their specific reactivity.

In detail, for *Chimer*_*Tb*, designed to target *T. brucei*, a statistically highly significant difference was observed in its reactivity between sera from animals infected with *T. congolense* and those infected with *T. brucei* (*P* = 0.0000). A relatively significant difference was also noted between the sera of animals infected with *T. vivax* and those infected with *T. brucei* (*P* = 0.0262). Finally, a statistically significant difference was established between *T. vivax* and *T. congolense* infections (*P* = 0.0000). These results suggest that *Chimer_Tb* can differentiate *T. brucei* from *T. congolense*, but its ability to discriminate *T. brucei* from *T. vivax* remains uncertain.

For *Chimer*_*Tc*, targeting *T. congolense*, a statistically significant difference was observed in its reactivity between sera from animals infected with *T. congolense* and those infected with *T. brucei* (*P* = 0.0001). The reactivity to *Chimer_Tc* between *T. vivax* and *T. brucei* infections was not statistically significant (*P* = 0.3040). However, a statistically significant difference was found between the sera of animals infected with *T. vivax* and those infected with *T. congolense* (*P* = 0.0044). These results suggest that *Chimer_Tc* can be a strong discriminator for *T. congolense*, despite some reactivity with *T. brucei* infection sera.

For *Chimer*_*Tv*, designed to target *T. vivax*, a statistically significant difference was observed in its reactivity between sera from animals infected with *T. congolense* and those infected with *T. brucei* (*P* = 0.0013). A statistically highly significant distinction was noted between the sera of animals infected with *T. vivax* and those infected with *T. brucei* (*P* = 0.0000), as well as between *T. vivax* and *T. congolense* infections (*P* = 0.0000). These results indicate that *Chimer_Tv* may have a preferential affinity for *T. vivax* infection sera.

Finally, for *Chimer_PanTryp-1*, designed as a pan-trypanosome antigen, statistically significant differences were observed in its reactivity between sera from animals infected with *T. congolense* and those infected with *T. brucei* (*P* = 0.0019). Similarly, a statistically significant difference was noted between sera from animals infected with *T. vivax* and those infected with *T. brucei* (*P* = 0.0009). The distinction between *T. vivax* and *T. congolense* infections also showed a statistically very significant difference (*P* = 0.0000). These data suggest that this chimera may be a relevant antigen for the detection of trypanosomosis in general, without clear species distinction on its own. These results likely reflect the expected cross-reactivity of a pan-trypanosome antigen, detecting infections broadly without clear species distinction.

## Discussion

The aim of the study was to design, produce and evaluate chimeric recombinant proteins for the serological diagnosis of African animal trypanosomoses (AAT), in order to overcome the constraints associated with the use of whole trypanosome lysates in antibody-ELISA, so far the serological test recommended by the WOAH.

Five chimeric proteins were designed. Two are genus-specific, or pan-trypanosome, aiming at detecting all African trypanosome infections. Three are species-specific, targeting *T. congolense* and *T. vivax*, the most pathogenic species of AAT, and *T. brucei* s.l. The later should detect not only *T.b. brucei*, but also other *Trypanozoon*, chiefly *T. evansi*, but also potentially *T.b. gambiense and T.b. rhodesiense*, if not *T. equiperdum*. The epitopes have been identified using rigorous criteria, including both bioinformatic prediction of the most antigenic regions and their conservation within the target trypanosome species. In contrast to most studies reviewed by (Gonçalves et al. 2024), we chose not to select only defined individual epitopes *per se* but rather larger exposed regions, potentially containing several epitopes. This choice favours a broader immune response and increases the sensitivity of the diagnostic test. Although this increases the overall size of the chimeras, it may also facilitate recombinant expression.

The five proteins have been successfully expressed as soluble protein in *E. coli* with yields ranging from 1 mg/L to 5 mg/L of culture, which can be considered an achievement, soluble production of eukaryotic proteins in bacteria, particularly artificial constructs, being particularly tricky (Pouresmaeil and Azizi-Dargahlou, 2023; Ojima-Kato, 2025). In this study, chimera expression was eased by using the pET-SUMO plasmid, a vector that is increasingly being used for recombinant protein production in *E. coli* (Munir et al. 2023). This system not only can lead to high-yield production, but also improves soluble expression and protein stability (Wang et al. 2010; Tan et al. 2020). It also facilitates purification using the 6xHis tag, which can subsequently be removed by enzymatic digestion. Purification was performed using a three-step protocol combining Ni-NTA affinity chromatography, ion-exchange chromatography on Q-sepharose, and size-exclusion chromatography on Sephadex 200. This protocol achieved high purity (>90%) while ensuring removal of the SUMO carrier fragment.

The five chimeras were tested in an antibody-ELISA using a panel of experimental infection sera. The availability of well-characterized sera remains a challenge due to the high cost of experimental infections, cold-storage constraints, and regulatory restrictions on sample movement (Nagoya protocol, biosecurity issue). Alternatives such as serum desiccation exist but are not widely implemented (Bossard and Desquesnes, 2023). For these reasons, the panel of sera used in the preliminary evaluation of the chimera’s diagnostic potential was relatively modest, and would not allow dependable calculation of test parameters. Regardless, we observed good reactivity with *Trypanosoma*-infected sera for four chimeras (*Chimer_Tb, Chimer_Tc, Chimer_Tv* and *Chimer_PanTryp-1*) compared to the corresponding total parasite lysates (Lysate_Tb, Lysate_Tc and Lysate_Tv) when using ELISA and Western blot for the serological detection of *T. brucei, T. congolense* and *T. vivax* infections. Longitudinal ELISA test results showed varied reactivity profiles for each antigen depending on the infecting species.

*Chimer_PanTryp-1* demonstrated high reactivity for *T. brucei* infection, surpassing the levels observed for Lysate_Tb and *Chimer_Tb*. For *T. vivax* infection, *Chimer_PanTryp-1* also showed high and rapid reactivity, as did Lysate_Tv. However, for *T. congolense* infection, although *Chimer_PanTryp-1* showed rapid initial reactivity, Lysate_Tc clearly outperformed this chimera in the advanced chronic phase. This suggests that while *Chimer_PanTryp-1* may be relevant in the early stages of infection, antibody persistence or diversity, as detected by total lysate, may be higher in the established or chronic phases of *T. congolense* infection. Nevertheless, this rapid antibody response kinetics are desirable for controlling parasitic diseases, particularly since animals in the advanced chronic phase are often treated or no longer present in the field (Maddison, 1991; Santano et al. 2022). Even more relevant, this could imply that the reactivity post-treatment would be lower than the current test, although it remains to be tested. Such rapid detection of this chimera could therefore be considered an active detection of infection (Fierz, 2004). The specific chimeras *Chimer_Tc* and *Chimer_Tv* exhibited high reactivity and distinct response kinetics for their respective target species. Although *Chimer_Tc* showed significant reactivity for *T. congolense*, Lysate_Tc outperformed it in the late phase. Similarly, *Chimer_Tv* displayed strong and rapid reactivity towards *T. vivax*, initially surpassing Lysate_Tv, which caught up at the end of the follow-up period. *Chimer_Tb*’s moderate reactivity for *T. brucei* was similar to that of Lysate_Tb, providing no significant improvement in detection levels.

The relative specificity of the chimeras was analysed to evaluate their ability to discriminate between different *Trypanosoma* species. *Chimer_Tb* demonstrated the capacity to distinguish *T. brucei* sera from *T. congolense* sera, though not significantly from *T. vivax* sera. *Chimer_Tc* demonstrated relative specificity for *T. congolense*, discriminating this infection from *T. brucei* and *T. vivax* infections. *Chimer_Tv* showed relative specificity for *T. vivax*, distinguishing this infection from *T. brucei* and *T. congolense* infections. However, caution is warranted given that the *T. vivax* and *T. brucei* sera are longitudinal, as they are from one and three animals, respectively, at different stages of infection. The results obtained with longitudinal sera from *T. congolense* are more reliable as these come from 24 animals. In addition, the *Chimer_Tb* Western blot showed recognition by both positive and negative controls, raising questions about the specificity of this chimera in this test format. This complicates its use for diagnosis and indicates the need for further optimization.

It is not clear why *Chimer_PanTryp-2* did not show any reactivity. Expression and purification were not different from the other chimeras, neither was the yield. Moreover, it encompassed epitopes from BiP/HSP70, which displayed a documented diagnosis potential (Boulangé et al. 2002; Bossard et al. 2010). On the positive side, the total absence of reactivity would indicate that the observed reactivity with the other chimeras is indeed specific and not an artefact of protein production or reactivity with a bacterial contaminant.

To summarize, while the preliminary findings suggest a good diagnostic potential of at least three out of five chimeras, *Chimer-Tb* remaining uncertain, expanded validation with a larger cohort is essential to solidify the observed specificity.

The next step will be to evaluate these antigens individually on (1) a broader panel of sera from experimental infections with *T. brucei, T. evansi, T. congolense* and *T. vivax*, (2) a large cohort of confirmed negative sera and (3) sera from non-trypanosome infections, in particular from tick-borne diseases, before validating the test on field samples, the whole alongside the WOAH recommended test. Testing human sera, at least on *Chimer_Tb* and *Chimer_PanTryp-1*, should also be endeavoured. Would the reactivity be confirmed, adaptation of this ELISA into a rapid test format could be a promising prospect, particularly for field diagnosis.

This work introduces a novel approach in the field of the diagnosis of trypanosomoses, where strategies using recombinant antigens usually rely on a single antigenic target. To our knowledge, this is one of the first studies to have designed and developed chimeric multivalent recombinant antigens specifically for this purpose.

## Data Availability

The data were deposited on the CIRAD Dataverse (France) (https://dataverse.cirad.fr/) and are available at Boulange, Alain, 2025, ‘Development of chimeric multivalent proteins for serological diagnosis of African animal trypanosomosis’ (https://doi.org/10.18167/DVN1/ZAN6XG).
